# Teaching Financial Fitness: Integrating a Financial Literacy Curriculum Into Radiation Oncology Residency Program

**DOI:** 10.7759/cureus.91913

**Published:** 2025-09-09

**Authors:** Afshin A Safa, P. Travis Courtney, Michael L. Steinberg

**Affiliations:** 1 Radiation Oncology, David Geffen School of Medicine, University of California Los Angeles, Los Angeles, USA; 2 Radiation Oncology, University of California Los Angeles, Los Angeles, USA

**Keywords:** business of medicine, patient financial toxicity, personal finance, physician financial literacy, radiation oncology residency education

## Abstract

Financial literacy remains a critical yet often neglected component of medical education. Radiation Oncology residents face significant financial stressors, including high educational debt, limited exposure to personal finance, and the increasing burden of financial toxicity among patients. These stressors can contribute to impaired decision-making from undue physician cognitive burden and subsequently, suboptimal patient care.

In response to these challenges, we developed a structured financial literacy curriculum for Radiation Oncology residents at the University of California, Los Angeles (UCLA). This initiative consists of two complementary components: (1) a six-lecture Personal Finance Curriculum repeated biennially, covering topics such as debt management, budgeting, investing, insurance, and career planning; and (2) a four-lecture Business of Medicine and Financial Toxicity Curriculum delivered annually, focused on healthcare reimbursement, insurance navigation, medical documentation, and financial toxicity in cancer care. Each lecture is accredited for one Continuing Medical Education (CME) credit, and the structure ensures that residents are exposed to each topic at least twice during their four-year radiation oncology training.

This curriculum directly addresses gaps in both physician financial literacy and the understanding of patient-level financial toxicity. By integrating behavioral finance principles, insurance literacy, and practice management skills, the program aims to reduce physician cognitive burden, improve financial decision-making, and enhance career satisfaction. Furthermore, by equipping residents with tools to help mitigate patient financial toxicity, the curriculum supports better patient outcomes and satisfaction. Integrating financial literacy into Radiation Oncology residency training is feasible, well-received, and urgently needed. The UCLA Financial Fitness Curriculum offers a scalable model that other training programs can adopt to improve financial decision-making by physicians, leading to better outcomes and satisfaction for patients.

## Introduction

Despite growing financial pressures on both physicians and patients, formal education in financial literacy remains largely absent from medical training, particularly in specialties like Radiation Oncology. To address this critical gap, we developed and implemented a structured financial literacy curriculum within the University of California, Los Angeles (UCLA) Radiation Oncology Residency Program, encompassing personal finance, the business of medicine, including the implications of value-based care models, and emphasizing strategies to mitigate financial toxicity for cancer patients. This report describes the structure, rationale, and potential impact of our curriculum, offering a replicable model for other residency programs.

Background

Radiation Oncology residents often face substantial educational debt yet lack knowledge about investment and retirement planning [1]. Financial literacy has been associated with improved financial planning behaviors and well-being [2-4]. Furthermore, physician financial stress has been linked to increased medical errors due to cognitive burden [5,6]. In addition, the rising prevalence of private equity acquisitions of physician practices has raised ethical concerns about the negative impact on both physician autonomy and the quality of patient care [7].

In parallel to physician-related concerns, the rising cost of healthcare has contributed to increased financial toxicity for patients [8]. Bankruptcies attributed to medical causes have risen significantly, negatively impacting patients [9]. Although radiation oncologists are increasingly aware of the financial burdens from cancer care their patients face, most do not routinely screen for patient-level financial toxicity, or engage in discussions about treatment costs [10,11]. This disconnect underscores another critical gap in radiation oncology training: the lack of understanding of the impact of financial aspects of cancer care delivery on the patient experience and outcomes. Left unaddressed, these financial burdens can lead to delays in care for patients and moral injury for clinicians. In response, we incorporated content into our financial literacy curriculum that covers the fundamentals of medical treatment reimbursements and navigating health insurance systems. The goal of this curriculum is to improve both residents’ understanding of personal finance as well as patient financial stability and satisfaction with cancer care. In view of the evolving reimbursement landscape, we highlight the shift toward value-based care, emphasizing how future income will be tied to outcomes and efficiency.

## Technical report

Curriculum development: adaptation of the money matters curriculum for radiation oncology training

The Money Matters Curriculum [12], described by Mizell et al. in 2014, consisted of 18 hours of instruction in financial literacy and practice management designed for surgical residents. We adapted its key principles to the UCLA Radiation Oncology Residency program, focusing on specific topics deemed most beneficial for Radiation Oncology residents. Additional material was incorporated from curricula developed by Johns Hopkins University Business School [3] and the University of North Carolina [6]. Our revised curriculum includes the following topics: 1. Student loan management: understanding repayment options, consolidation, and forgiveness programs such as Public Service Loan Forgiveness (PSLF), given the high levels of educational debt [2,3]. 2. Budgeting (income and expense management): money basics and skills to create and maintain a budget to manage their finances effectively and reduce financial stress [2,4]. 3. Retirement planning: understanding retirement savings options, including 401(k), 403(b), and Individual Retirement Arrangements (IRAs), compound interest, and the importance of early contributions [2,3]. 4. Personal insurance: understanding the need for and types of personal insurance, including disability and life insurance, is essential for protecting against unforeseen risks [12]. 5. Financial advisors: How and when to select and interact with advisors while avoiding medical scams, unnecessary fees, and commissions [6]. 6. Investment strategies: basic principles of investing, risk assessment, diversification, and different investment vehicles like mutual funds, stocks, bonds, and real estate [2,4]. 7. Tax planning: basic tax principles, deductions, strategies to minimize tax liabilities, estate planning, and asset protection, enabling residents to navigate tax obligations effectively [12]. 8. Practice management: Learning the financial aspects of running a medical practice, billing, coding, insurance authorization appeals, contract negotiation, and efficiency in building and maintaining practice [12].

Curriculum structure: personal finance module

The Personal Finance curriculum consists of six lectures (Table 1), each accredited for one Continuing Medical Education (CME) credit. Lectures are delivered on a two-year repeating cycle, ensuring each resident the opportunity to attend each lecture at least twice during their four-year radiation oncology residency. This format reinforces key financial concepts and supports progressive skill development.

**Table 1:** Personal finance module lecture topics

Week	Topic A	Topic B
1	Personal Finance Fundamentals:	Personal Insurance Fundamentals:
	Budgeting and expense management	Insurance essentials: Health, disability, life and property
	Fundamentals of retirement savings	Health Maintenance Organizations (HMO) vs. Preferred Provider Organizations (PPO)
	Wonder of compound interest with early contributions	Health Savings Accounts (HSA) vs.Flexible Spending Accounts (FSA)
2	Debt Management:	Credit Strategy:
	Strategies for education student loans: forgiveness, refinancing, consolidation	Building, maintaining and protecting credit scores
	Budgeting tools	Responsible use of credit
	Financial habits	Debt reduction strategies
3	Investment Strategies:	Tax Planning:
	Principles of investing: Risk vs. return	Roth accounts
	Active vs. passive investment strategies	Tax implications of retirement accounts
	Diversification: mutual funds vs. index funds	Tax deductions
	Retirement account structures	Strategies to minimize tax liability
4	Asset Protection:	Estate Planning:
	Annuity insurance	Wills vs.trusts
	Pensions	Power of attorney
	Social security	Asset transfer
5	Career Planning:	Financial Scams:
	Contract negotiation of compensation packages	Avoiding hidden financial scams
	Office operational efficiency	Predatory financial "advisors"
	Practice Management Pearls	Avoiding hidden fees
6	Behavioral Finance:	Long-Term Financial Planning for Physicians:
	Psychological biases in decision-making	Creating a personal plan aligned with life goals
	Financial goal setting	Maintaining intentional living

Learning Objectives and Rationale: Personal Finance Module 

The primary objective of this curriculum is to equip Radiation Oncology residents with essential financial skills. Debt and credit management are significant sources of stress for trainees and physicians. By addressing behavioral finance principles, residents learn to identify and avoid common decision-making pitfalls that can lead to poor financial outcomes. They also develop a solid understanding of investment vehicles, their tax implications, asset protection strategies, estate planning, and insurance options, all of which support long-term financial independence. Additionally, practical guidance on contract negotiation and career planning empowers residents to make informed career choices. As a result, physicians who are not burdened by financial stress due to limited financial literacy may experience reduced cognitive load, which contributes to fewer medical errors and improved patient outcomes [5,6]. 

Curriculum structure: business of medicine and financial toxicity module

Taught in tandem with the Personal Finance Module, this curriculum consists of four lectures (Table 2) accredited for one CME credit, repeated annually, ensuring residents have at least four opportunities to attend each session during their residency. Residents learn to manage the often-overlooked business aspects of medicine, with a focus on mitigating financial toxicity for cancer patients and navigating the complex healthcare reimbursement challenges.

**Table 2:** Business of medicine and financial toxicity module lecture topics

Week	Topic
1	Business of Medicine:
	International Classification of Diseases (ICD) introduction
	Current Procedural Terminology (CPT) coding
	Evaluation and Management (E/M) documentation pitfalls
	Understanding Relative Value Units (RVUs)
	Radiation Oncology Business Models (ROBM)
2	Process of Care in Radiation Oncology:
	Clinical treatment planning
	Simulation and treatment planning
	Treatment delivery
	Weekly management
3	Preventing Financial Toxicity for Patients:
	Medical necessity
	Insurance authorization process
	Insurance Basics: Medicare, Medicaid and PPOs
	Contract negotiations
	Reimbursement models
	Denial appeal process
4	Malpractice and Defensive Medicine:
	Case studies on denials
	Medico-legal challenges
	Legislative updates on cancer care reimbursement
	Review of American Society for Radiation Oncology (ASTRO) guidelines
	Review of American College of Radiology (ACR) guidelines

Learning Objectives and Rationale: Business of Medicine and Financial Toxicity Module

The primary objective of this curriculum is to develop financial fluency in medical coding, documentation, and administrative workflows. When physicians understand these systems, they are better positioned to identify strategies to reduce financial toxicity for their patients. Proficiency in navigating insurance processes such as denials, prior authorizations, and appeals can help minimize delays in care due to insurance delays, ultimately leading to improved patient outcomes and satisfaction while reducing moral injury to physicians. Additionally, by recognizing legal risks in medical coding and documentation and applying judicial defensive medicine principles, both the quality and safety of care are improved.

Lectures are delivered in a hybrid format by national leaders in billing, coding, and finance. Guest lecturers from UCLA and Johns Hopkins business schools also contribute. Attendance is voluntary, with consistently high engagement and average ratings of 4.5/5. Anonymous post-lecture evaluations and informal surveys indicate strong engagement and perceived value. We plan to conduct a formal evaluation via pre- and post-questionnaire surveys to assess knowledge acquisition. 

In addition to personal financial literacy, future professional income for radiation oncologists will increasingly depend on their ability to deliver high-quality care within value-based reimbursement structures. Understanding bundled payment models and hypofractionation strategies is essential for aligning clinical decisions with institutional financial sustainability. For example, to illustrate bundled payment dynamics and the curriculum’s relevance, please refer to Table 3, which maps revenue drivers in value-based care.

**Table 3:** Mapping curriculum topics to revenue drivers in value-based care

Curriculum Topic	Revenue Driver	Example
Hypofractionation	Reduced treatment fractions, Bundle payment models	Increased capacity, lower cost
Documentation	Avoidance of denials	Accurate E/M coding
Insurance Navigation	Timely authorization	Reduced delays in care

## Discussion

Financial literacy improves physician financial decision-making and improves patient satisfaction and outcomes, yet few training programs offer it. The integration of financial literacy into medical training has yielded positive outcomes in other residency programs. Internal Medicine programs at Stony Brook University Hospital and Johns Hopkins Bayview Medical Center have successfully implemented financial literacy programs, including biannual financial planning sessions [3,13]. Surgical and neurosurgical residency programs have benefited from curricula combining practice management and personal finance to enhance residents’ financial competence and career readiness [14]. Understanding repayment options for student loans, budgeting, retirement savings, and investment strategies are crucial for managing finances effectively [2-4].

Financial literacy has been shown to enhance patient care by alleviating the emotional and cognitive burdens that financial stress places on residents [5,6]. This financial strain is a known contributor to emotional exhaustion and depersonalization, factors that can compromise the quality of care. Moreover, higher educational debt has been linked to lower scores on medical knowledge assessments, highlighting the cognitive toll of financial insecurity [15]. Equipping radiation oncology residents with financial literacy is, therefore, crucial - not only to optimize physician decision making and future professional career sustainability, but also to reduce the financial toxicity experienced by cancer patients, ultimately leading to greater patient satisfaction and improved quality of care.

This initiative focuses on both personal financial literacy and the business side of medicine and aims to reduce financial toxicity for cancer patients, particularly in the context of insurance denials, by empowering residents with the knowledge and tools to navigate these challenges and better advocate for their patients. Anecdotally, this program has been well received. Moving forward, we plan to expand the series with additional lectures that address the broader financial challenges associated with the high costs of cancer treatment, further supporting both resident physicians and cancer patients, especially those facing financial hardship. Additionally, we plan to collect survey data to more formally evaluate the program’s impact and content.

## Conclusions

This curriculum demonstrates the feasibility of implementing structured financial education in residency training, with preliminary evidence of improved self-reported financial literacy. By integrating comprehensive financial education into our radiation oncology training program, we hope to empower residents to confidently manage their personal and professional finances, navigate the complexities of the healthcare system, and minimize the financial toxicity experienced by cancer patients. Future studies will evaluate the curriculum’s impact on decision-making, stress reduction, and patient outcomes in value-based care using validated survey instruments. We hope that the benefit of this curriculum extends beyond financial security by ultimately enhancing both patient outcomes and satisfaction. As financial pressures on physicians and patients continue to intensify, it is essential that residency programs across all specialties adopt similar initiatives. Now is the time to ensure that every medical resident is equipped with the financial knowledge needed to thrive both personally and professionally.
